# Supplementary material for: Global COI meta-analysis reveals ocean-basin genetic structure in Sphyrna lewini
Source: PLoS One. 2026 Mar 20;21(3):e0344911. doi: 10.1371/journal.pone.0344911 (PMC13004374; doi:10.1371/journal.pone.0344911)
Supplement: S1 File — (DOCX) [file pone.0344911.s001.docx]

## Supplemental Figures


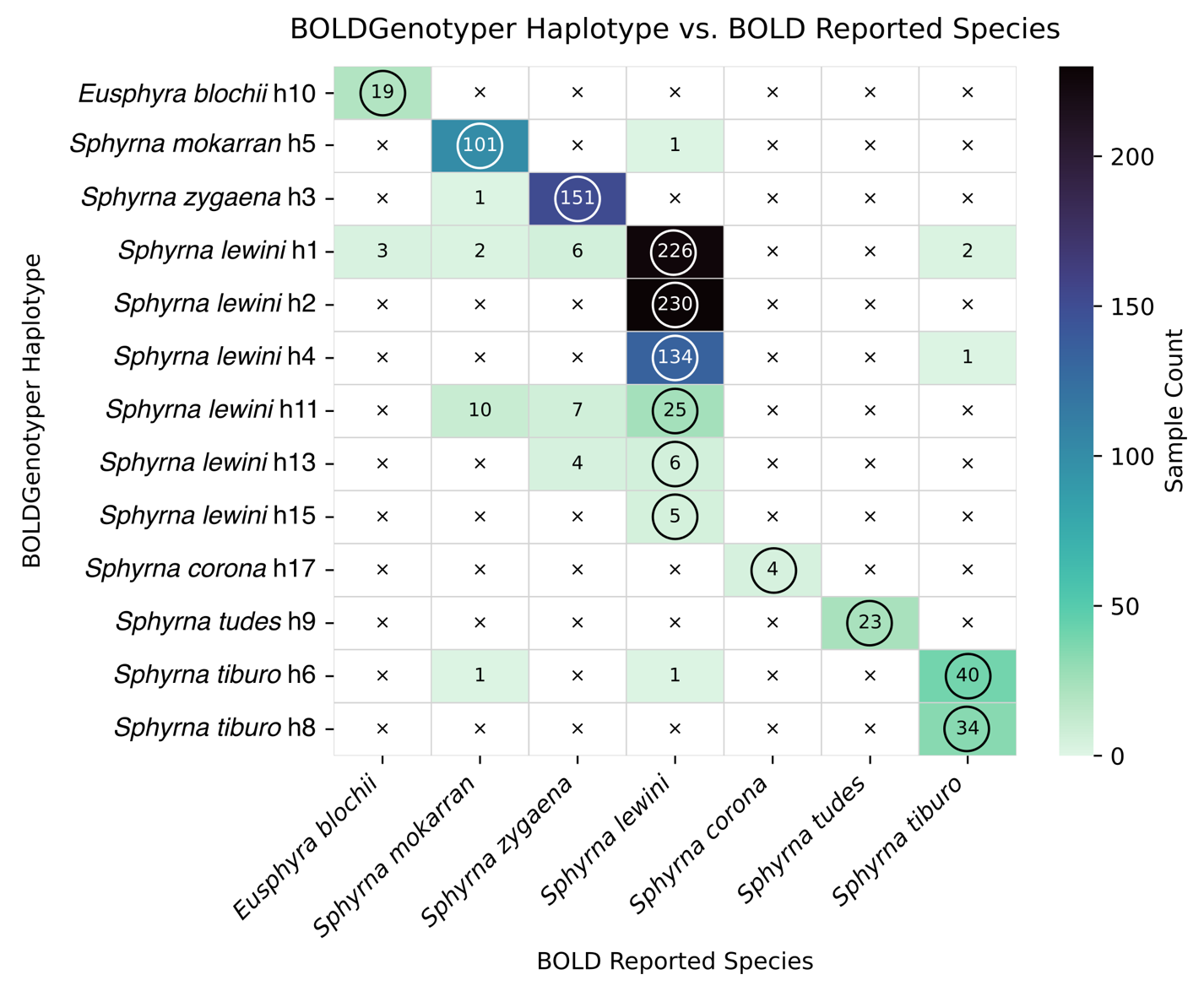
**Figure S1. Concordance between COI haplotype assignments and BOLD-reported species labels for all Sphyrnidae samples.** Heatmap showing the concordance between each COI haplotype identified here (rows) and the species name reported in BOLD (columns). Cell shading represents sample count, with darker colors indicating higher abundance. Crosses indicate haplotype-species combinations for which no samples were present.


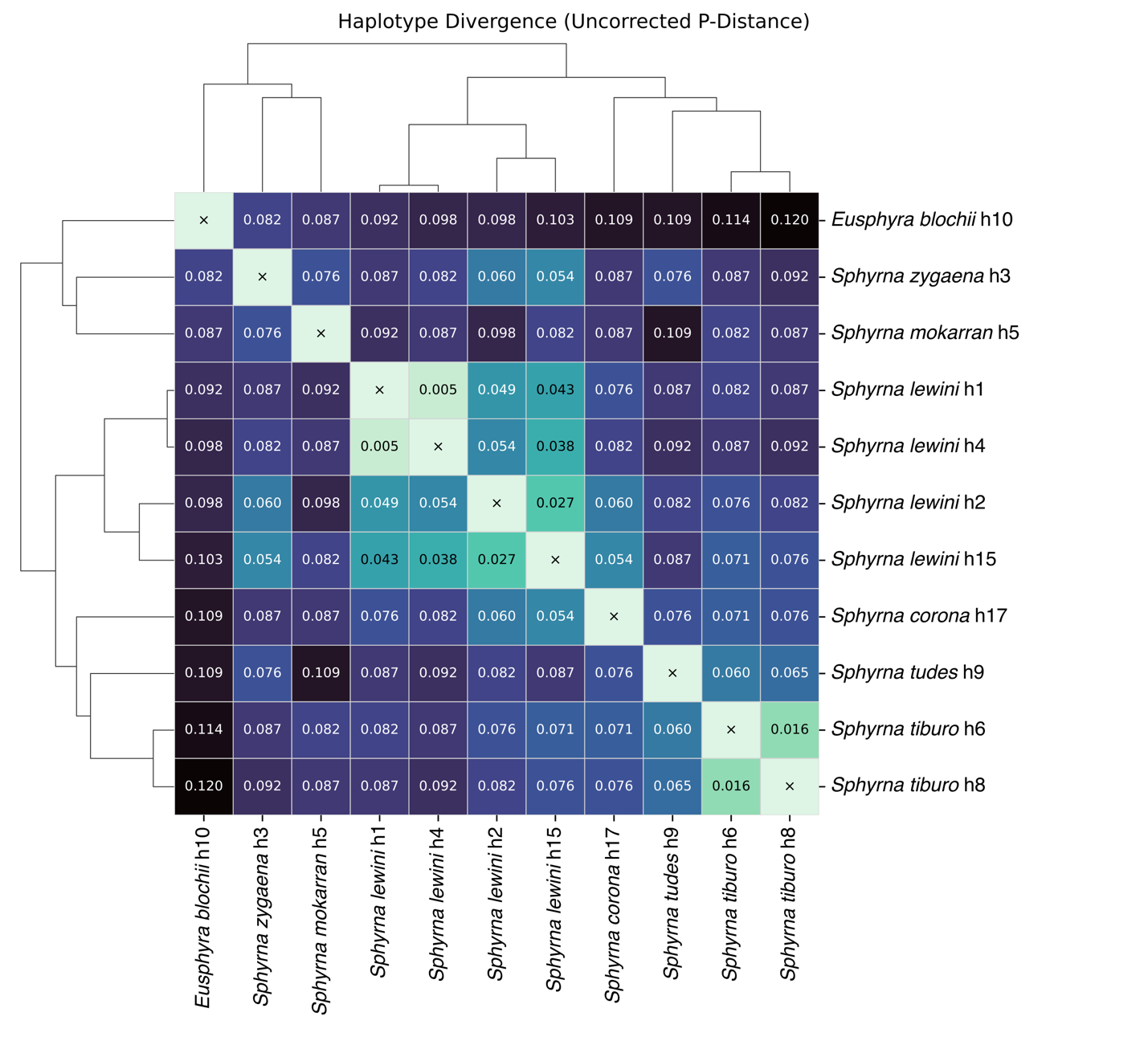


Figure S2. Pairwise uncorrected p-distance heatmap showing sequence divergence among all Sphyrnidae COI haplotypes. Heatmap of uncorrected pairwise p-distances calculated from the 184 bp COI fragment alignment, with hierarchical clustering applied to rows and columns to visualize similarity among haplotypes. Lower distances (light colors) indicate high sequence similarity, whereas higher distances (dark colors) reflect greater divergence.
