# Supplementary material for: Global COI meta-analysis reveals ocean-basin genetic structure in Sphyrna lewini
Source: PLoS One. 2026 Mar 20;21(3):e0344911. doi: 10.1371/journal.pone.0344911 (PMC13004374; doi:10.1371/journal.pone.0344911)
Supplement: Attachment — Submitted filename: Review - PONE-D-25-65513.pdf [file pone.0344911.s003.pdf]

Review PONE-D-25-65513
Global COI meta-analysis reveals ocean-basin genetic structure in *Sphyrna lewini*

This manuscript presents a global meta-analysis of publicly available COI sequences to investigate ocean-basin–scale genetic structure in *Sphyrna lewini*. The study addresses a timely and relevant topic, given the species' current Critically Endangered status, and demonstrates the utility of family-level comparative analyses for identifying identification inconsistencies in public databases. The overall approach is sound, and the results have clear implications for conservation and management. The following comments are intended to improve clarity, taxonomic context, and the presentation of the manuscript.

Introduction

The Introduction provides limited taxonomic information on the family Sphyrnidae, the genera (*Eusphyra* and *Sphyrna*), and the focal species. In addition, it does not sufficiently address the taxonomic issues currently recognized within the genus *Sphyrna*.

Line 87: In objective (1), replace "hammerhead" with "Sphyrnidae".

Suggested revision:
Evaluate the extent of taxonomic inconsistencies in BOLD Sphyrnidae records through comparative analysis.

Results

The tables presented in the text should be properly formatted.

Discussion

Line 179:
"The morphological similarity among hammerhead species, particularly between *S. lewini*, *S. zygaena*, and *S. mokarran*, likely contributes to field identification errors that propagate into sequence databases."

I suggest removing "particularly between *S. lewini*, *S. zygaena*, and *S. mokarran*", as these species do not exhibit close morphological similarity.

Suggested revision:
The morphological similarity among hammerhead species likely contributes to field identification errors that propagate into sequence databases.
